# Novel insights into biosynthesis and uptake of rhamnolipids and their precursors

**DOI:** 10.1007/s00253-016-8041-3

**Published:** 2016-12-17

**Authors:** Andreas Wittgens, Filip Kovacic, Markus Michael Müller, Melanie Gerlitzki, Beatrix Santiago-Schübel, Diana Hofmann, Till Tiso, Lars Mathias Blank, Marius Henkel, Rudolf Hausmann, Christoph Syldatk, Susanne Wilhelm, Frank Rosenau

**Affiliations:** 1 Ulm Center for Peptide Pharmaceuticals (U-PEP), Ulm University, Albert-Einstein-Allee 11, 89081 Ulm, Germany; 2 Institute for Molecular Enzyme Technology (IMET), Heinrich-Heine-University Düsseldorf, Forschungszentrum Jülich, Wilhelm-Johnen-Straße, 52428 Jülich, Germany; 3 Boehringer Ingelheim Pharma GmbH & Co. KG, Biopharmaceutical and Analytical Development, Birkendorfer Straße 65, 88400 Biberach an der Riß, Germany; 4 Institute of Process Engineering in Life Sciences, Section II: Technical Biology, Karlsruhe Institute of Technology (KIT), Engler-Bunte-Ring 1, 76131 Karlsruhe, Germany; 5 Central Institute for Engineering, Electronics and Analytics, Section Analytics (ZEA-3), Forschungszentrum Jülich, Wilhelm-Johnen-Straße, 52428 Jülich, Germany; 6 Institute for Bio- and Geosciences, IBG-3: Agrosphere, Forschungszentrum Jülich, Wilhelm-Johnen-Straße, 52428 Jülich, Germany; 7 Institute of Applied Microbiology (iAMB), Aachen Biology and Biotechnology (ABBt), RWTH Aachen University, Worringerweg 1, 52074 Aachen, Germany; 8 Institute of Food Science and Biotechnology, Department of Bioprocess Engineering (150k), University of Hohenheim, Fruwirthstraße 12, 70599 Stuttgart, Germany; 9 iQu Collegiate-Didactics, Heinrich-Heine-University Düsseldorf, Universitätsstraße 1, 40225 Düsseldorf, Germany

**Keywords:** *Pseudomonas aeruginosa*, Rhamnolipids, Biosurfactant, *Pseudomonas putida*, Biosynthesis pathway

## Abstract

The human pathogenic bacterium *Pseudomonas aeruginosa* produces rhamnolipids, glycolipids with functions for bacterial motility, biofilm formation, and uptake of hydrophobic substrates. Rhamnolipids represent a chemically heterogeneous group of secondary metabolites composed of one or two rhamnose molecules linked to one or mostly two 3-hydroxyfatty acids of various chain lengths. The biosynthetic pathway involves rhamnosyltransferase I encoded by the *rhlAB* operon, which synthesizes 3-(3-hydroxyalkanoyloxy)alkanoic acids (HAAs) followed by their coupling to one rhamnose moiety. The resulting mono-rhamnolipids are converted to di-rhamnolipids in a third reaction catalyzed by the rhamnosyltransferase II RhlC. However, the mechanism behind the biosynthesis of rhamnolipids containing only a single fatty acid is still unknown. To understand the role of proteins involved in rhamnolipid biosynthesis the heterologous expression of *rhl*-genes in non-pathogenic *Pseudomonas putida* KT2440 strains was used in this study to circumvent the complex *quorum sensing* regulation in *P*. *aeruginosa*. Our results reveal that RhlA and RhlB are independently involved in rhamnolipid biosynthesis and not in the form of a RhlAB heterodimer complex as it has been previously postulated. Furthermore, we demonstrate that mono-rhamnolipids provided extracellularly as well as HAAs as their precursors are generally taken up into the cell and are subsequently converted to di-rhamnolipids by *P*. *putida* and the native host *P*. *aeruginosa*. Finally, our results throw light on the biosynthesis of rhamnolipids containing one fatty acid, which occurs by hydrolyzation of typical rhamnolipids containing two fatty acids, valuable for the production of designer rhamnolipids with desired physicochemical properties.

## Introduction

The biosurfactant rhamnolipid, first described by Jarvis and Johnson (1949), has various physiological roles and industrial applications (Lang and Wullbrandt 1999; Maier and Soberón-Chávez 2000). Rhamnolipids are produced and secreted to the extracellular milieu mainly by bacteria of the genus *Pseudomonas* (Abdel-Mawgoud et al. 2010). The opportunistic human pathogen *Pseudomonas aeruginosa* is among the best rhamnolipid producers (Giani et al. 1997; Müller et al. 2011), although bacteria from the genus *Burkholderia* also produce rhamnolipids (Häußler et al. 1998; Andrä et al. 2006; Funston et al. 2016).

In *P*. *aeruginosa*, rhamnolipids are essential for swarming motility, involved in biofilm formation and act as hemolysins (Köhler et al. 2000; Davey et al. 2003; Tremblay et al. 2007) what makes them to important virulence factors (Kownatzki et al. 1987). Rhamnolipids play a role in shielding of *P*. *aeruginosa* cells from host defense, e.g., they inhibit the phagocytosis by macrophages (McClure and Schiller 1996; van Gennip et al. 2009; Alhede et al. 2009). Additionally, rhamnolipids enhance the uptake of hydrophobic substrates like long-chain alkanes, e.g., octadecane (Zhang and Miller 1995; Al-Tahhan et al. 2000; Noordman and Jassen 2002).

Rhamnolipids feature a low toxicity and an enhanced biodegradability in comparison to detergents with petrochemical origin (Maslin and Maier 2000; Johann et al. 2016). Based on their surface active properties, they are used for bioremediation (Nguyen et al. 2008), enhanced oil recovery (Wang et al. 2007), and in cosmetic and food industries (Banat et al. 2010).

**Fig. 1:**
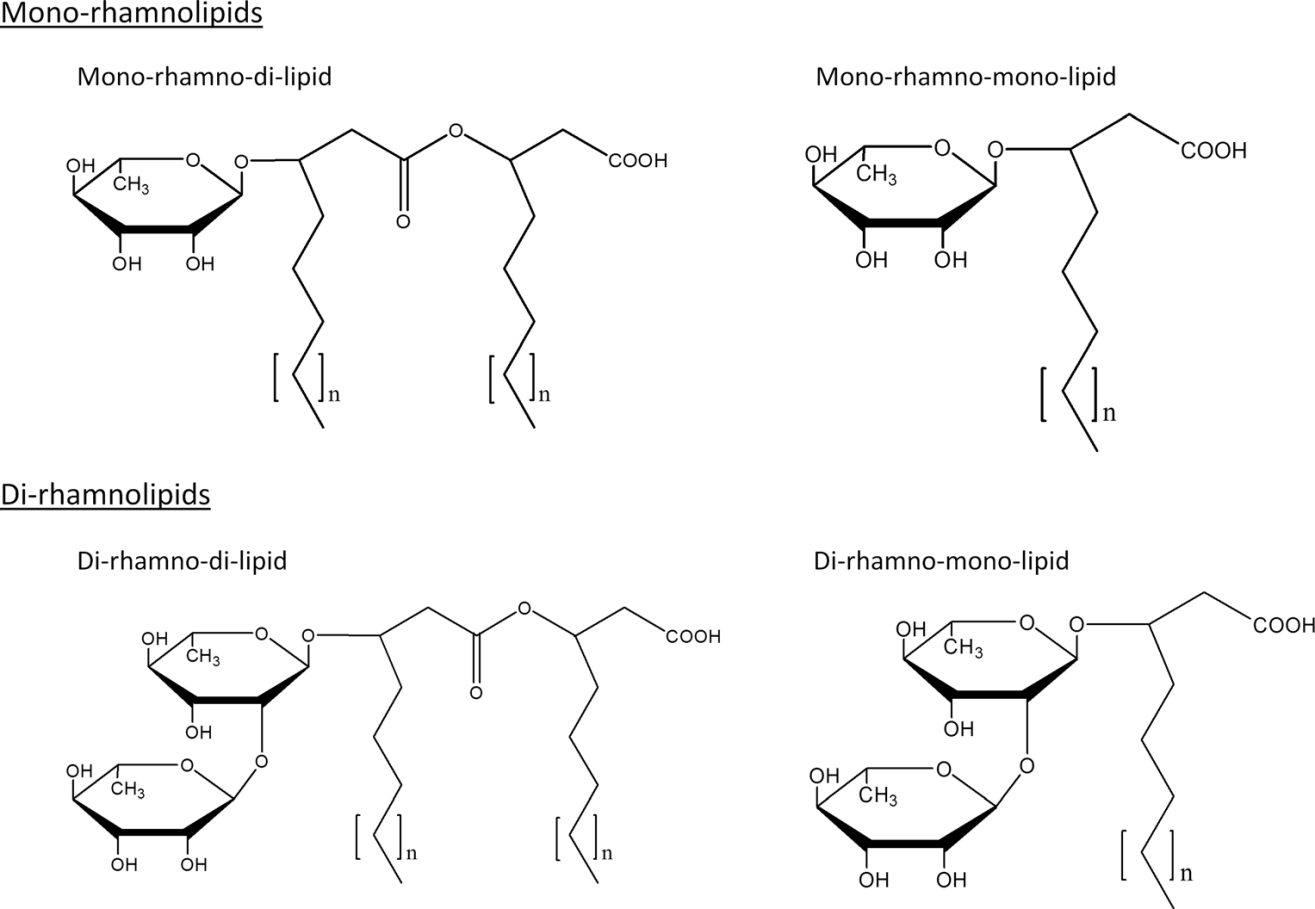
*Chemical* *structures of rhamnolipids*. Rhamnolipids are separated into mono- and di-rhamnolipids based on the number of L-rhamnose residues. Beside typical rhamnolipid species containing two 3-hydroxyfatty acids (mono-rhamno-di-lipid and di-rhamno-di-lipid), there exist species containing only one fatty acid chain (mono-rhamno-mono-lipid and di-rhamno-mono-lipid). Rhamnolipids from *P. aeruginosa* typically contain fatty acids with chain lengths between C_8_ and C_14_ (*n* = 1–7) while organisms from the genus *Burkholderia* produce rhamnolipids with longer alkyl chains and typical lengths between C_12_ and C_16_ (*n* = 5–9)

Rhamnolipids belong to the chemically diverse group of glycolipids composed of a hydrophilic rhamnose sugar moiety, which is linked through a β-glycosidic bond to a hydrophobic fatty acid moiety (Hauser and Karnovsky 1957). The number of rhamnose molecules allows a systematic distinction between mono- and di-rhamnolipids. The fatty acid residue typically consists of a dimer of two 3-hydroxyfatty acids forming an intramolecular ester (Déziel et al. 1999; Abdel-Mawgoud et al. 2010), although rhamnolipids with only one 3-hydroxyfatty acid are known, too (Syldatk et al. 1985a). These rhamnolipids are called mono-rhamno-mono-lipids and di-rhamno-mono-lipids, respectively (Fig. 1). These four species show different physicochemical properties, whereby they can be selectively used for various applications.

In *P. aeruginosa*, the length of the fatty acid chains in rhamnolipids varies from C_8_ to C_14_ with a predominant species containing C_10_-C_10_ fatty acid chains in mono- as well as in di-rhamnolipids (Déziel et al. 1999; Abdel-Mawgoud et al. 2010). In contrast, rhamnolipids produced by bacteria from the genus *Burkholderia* contain long-chain fatty acid with a predominant C_14_-C_14_ species (Manso Pajarron et al. 1993; Dubeau et al. 2009). Additionally, mono- and di-unsaturated fatty acids can be found in rhamnolipids, further expanding the existing chemical diversity of rhamnolipids (Abalos et al. 2001) and their potential applications in biotechnology and industry.

Rhamnolipid biosynthesis occurs in three consecutive enzymatic reactions. In the first step, RhlA synthesizes 3-(3-hydroxyalkanoyloxy)alkanoic acids (HAAs) by esterification of two 3-hydroxyacyl molecules bound to acyl carrier protein (ACP) descending from the fatty acid de novo synthesis (Rehm et al. 2001). RhlB links an HAA molecule with dTDP-L-rhamnose descending from glucose-6-phosphate (Olvera et al. 1999; Rahim et al. 2000), to create mono-rhamnolipids (Ochsner et al. 1994a). The last reaction in the di-rhamnolipid synthesis pathway is catalyzed by the rhamnosyltransferase II (RhlC), which joins a second dTDP-L-rhamnose molecule to the mono-rhamnolipids (Rahim et al. 2001). The biosynthesis of mono- and di-rhamno-mono-lipids with a single 3-hydroxyfatty acid is still speculative. Possibly, they descend from direct condensation of a dTDP-L-rhamnose with a 3-hydroxyfatty acid chain by RhlB. These molecules could be used as precursors for synthesis of di-rhamno-mono-lipids by RhlC. Optionally, they could be produced by hydrolysis of one unit from the dimer of esterified fatty acids in mono- and di-rhamno-di-lipids by a still unknown enzyme (Soberón-Chávez et al. 2005).

The genes *rhlA* and *rhlB* are organized in a bicistronic operon and encode proteins originally described as two subunits forming a functional rhamnosyltransferase I enzyme complex (Ochsner et al. 1994a). However, evidences that a *P*. *aeruginosa* Δ*rhlB* mutant strain produces HAAs (Déziel et al. 2003) and that heterologous expression of *rhlA* in *Escherichia coli* leads to production of HAAs (Zhu and Rock 2008) indicate that RhlA exerts its function independently of RhlB. The gene *rhlC* is also organized in a bicistronic operon with *PA1131*, a gene of unknown function (Rahim et al. 2001).

In *P. aeruginosa*, the two *rhl*-operons are transcriptionally regulated by the *q* *uorum sensing* (QS) regulatory network (Ochsner et al. 1994b; Ochsner and Reiser 1995; Pearson et al. 1997) and probably by other signaling systems (Wilhelm et al. 2007; Rosenau et al. 2010; Henkel et al. 2013). The QS autoinducer molecules butanoyl-homoserine-lactone (C_4_-HSL) and 3-oxo-dodecanoyl-homoserine-lactone (3-oxo-C_12_-HSL) are synthesized by RhlI and LasI, respectively. After the concentration of autoinducer molecules reaches a threshold, they bind to the regulator proteins RhlR and LasR to induce the expression of the *rhl*-genes (Williams and Cámara 2009; Reis et al. 2011).

The complex regulatory network controlling the rhamnolipid production in *P*. *aeruginosa* and its classification as an opportunistic pathogen are bottlenecks for their production (Müller and Hausmann 2011) and disadvantageous for many industrial applications (Toribio et al. 2010). Searching for an alternative rhamnolipid producing host, the non-pathogenic *Pseudomonas putida* KT2440 strain was identified as a suitable organism (Ochsner et al. 1995; Wittgens et al. 2011; Behrens et al. 2016; Beuker et al. 2016). It provides both pathways essential for the production of 3-hydroxyfatty acids and dTDP-L-rhamnose used as rhamnolipid precursors (Nelson et al. 2002). Although evolutionary closely related with *P. aeruginosa*, *P. putida* is lacking the complex regulatory circuits found in *P*. *aeruginosa*, what makes *P. putida* KT2440 a favorable host for the heterologous production of rhamnolipids and an ideal simplified genetic and physiologic background to study molecular aspects of rhamnolipid biosynthesis.

In this study, we have expressed *P*. *aeruginosa* genes involved in rhamnolipid biosynthesis in *P*. *putida* KT2440 to address the question if their protein-protein interactions play a role for function and/or stabilization of individual enzymes. A set of expression plasmids containing single genes or operons was used for modular expression of different gene combinations. Chemical analysis of produced rhamnolipids and precursors revealed novel insights in molecular interactions of RhlA and RhlB. Furthermore, we could show that exogenous rhamnolipids and HAAs are taken up by the cell and that they flow into the rhamnolipid biosynthesis pathway. These novel insights in the rhamnolipid biosynthesis pathway can be used for modulation of biochemical properties to create designer rhamnolipids.

## Material and methods

### Bacterial strains and culture conditions

*P*. *aeruginosa* PAO1 (DSM-22644; Hancock and Carey 1979), *P*. *putida* KT2440 (DSM-6125; Nelson et al. 2002), and *E*. *coli* DH5α (DSM-6897; Grant et al. 1990) were routinely cultivated in 10 ml LB medium (10 g/l tryptone, 5 g/l yeast extract, 10 g/l NaCl) in 100 ml Erlenmeyer flasks. All strains were grown at 150 rpm orbital shaking and 37 °C except *P. putida*, which were cultivated at 30 °C.

### Amplification of *rhl*-genes and plasmid construction

The genes for rhamnolipid biosynthesis were amplified as single genes or operon structures from the genomic DNA of *P*. *aeruginosa* PAO1 as a template (isolated with DNeasy Blood and Tissue Kit, QIAGEN, Hilden, Germany) using *Pfu*Turbo DNA polymerase (Stratagene, Waldbronn, Germany) as described by the supplier. The single genes were amplified starting 20 bp upstream of the start codon to maintain the native ribosomal binding site. The *rhlAB* and *PA1131-rhlC* operons started at their native transcriptional start sequences (see Rahim et al. 2001) to maintain the original full length untranslated regions (5´-UTR), which for *rhlAB* resulted in the highest amounts of mono-rhamnolipids in previous experiments comparing various lengths of upstream regions (data not shown). The sequences of the primers, obtained from Eurofins MWG Operon (Ebersberg, Germany), and restriction sites used for cloning of the resulting PCR products are listed in Table 1. The hydrolyzed PCR products were ligated into the respective sites of the pVLT33 vector (de Lorenzo et al. 1993) using restriction enzymes and T4 DNA ligase (Fermentas GmbH, St. Leon-Rot, Germany) as recommended by the supplier. DNA recombination was carried out as described in Sambrook and Russell (2001). *E*. *coli* DH5α cells were transformed with the resulting recombinant plasmids (Table 1) using standard protocol (Hanahan 1983) and positive clones were selected on LB-agar containing 50 μg/ml kanamycin after their incubation over night at 37 °C. The expression plasmid pVLT33_*rhlABC* containing the biosynthetic operon *rhlABC* was created by cloning an additional *rhlC* gene into pVLT33_*rhlAB* (Table 1).

**Table 1:** PCR primers, restriction enzymes and resulting recombinant plasmids or *P. aeruginosa* mutant strains

Gene/operon	Primer	Sequence (5′- > 3′)	Restriction enzymes	Recombinant plasmid or mutant strain
*rhlA*	Up	TTGAATTCAAATTTTTGGGAGGTGTGAAATGCGGCG	*Eco*RI	pVLT33_*rhlA*
	Down	TTTGGTACCTCAGGCGTAGCCGATGGCC	*Acc*65I	
*rhlB*	Up	TTTGGTACCATAACGCACGGAGTAGCCCCATGC	*Acc*65I	pVLT33_*rhlB*
	Down	TTTTTCTAGATCAGGACGCAGCCTTCAGCC	*Xba*I	
*rhlC*	Up	TTTTTCTAGACCTACGGGAGAAGAACGATCATGGACCG	*Xba*I	pVLT33_*rhlC*
	Down	TTTAAGCTTCTAGGCCTTGGCCTTGCCGG	*Hin*dIII	
*rhlAB*	Up	TTGAATTCCATCGGCTACGCGTGAACACGG	*Eco*RI	pVLT33_*rhlAB*
	Down	TTTTTCTAGATCAGGACGCAGCCTTCAGCC	*Xba*I	
*PA1131-rhlC*	Up	TTTTTCTAGAAGGATTTCCTGTGTTCGCCGGGAG	*Xba*I	pVLT33_*PA1131*-*rhlC*
	Down	TTTAAGCTTCTAGGCCTTGGCCTTGCCGG	*Hin*dIII	
rhlA*B	Up	TGGTCTCCGCGGCCTGGGGCGGT^a^	–	pVLT33_rhlA*B
	Down	ACCGCCCCAGGCCGCGGAGACCA^a^	–	
additional cloning of *rhlC* in pVLT33_*rhlAB*			As above	pVLT33_*rhlABC*
*rhlA*-up	Up	TTTGACTCCCCGTCGACACCCTCCATGACCATCAAATCGGACAAG	*Ahd*I	*P. aeruginosa* Δ*rhlA*
	Down	AAACAATTGTTCACACCTCCCAAAAATTTTCGAACAGGCAAAC	*Mun*I	
*rhlA*-dn	Up	AAACAATTGACCCTTGACCTGCGAAGACCCG	*Mun*I	*P. aeruginosa* Δ*rhlA*
	Down	AAATTAATAAGGCTCCCAGTGGCGCG	*Ase*I	
*rhlC*-up	Up	TTTGACTCCCCGTCCCGTCCTGGTCCTGGCGATGC	*Ahd*I	*P. aeruginosa* Δ*rhlC*
	Down	TTTCAATTGGTCTATCCGGTCCATGATCGTTCTTCTCCCG	*Mun*I	
*rhlC*-up	Up	AAACAATTGTAGTCGGCGAAACGCATTCCCGC	*Mun*I	*P. aeruginosa* Δ*rhlC*
	Down	AAATTAATGGCGCTTCACCGAGGCGTATCC	*Ase*I	

^a^Underlined GCC codon was used for S102A exchange of RhlA

### Structure homology modeling and site-directed mutagenesis of the *rhlA* gene

The three-dimensional structure of RhlA was modeled using the Phyre server (Kelley and Sternberg 2009) with *Streptomyces lividans* chloroperoxidase L (PDB code: 1A88) identified as the best template, with a 14% sequence identity, an E-value of 1 × 10^−35^ and a 100% prediction confidence. Chloroperoxidase L belongs to the family with an α/β-hydrolase fold and the Ser-His-Asp catalytic triad. The structural superimposition of a RhlA model and a chloroperoxidase L structure was performed using the PyMOL software (DeLano 2002). To inactivate, RhlA Ser102, identified as a catalytic residue of RhlA, was replaced by alanine with the QuikChange® Site-Directed Mutagenesis Kit (Stratagene, Waldbronn, Germany) using the plasmid pVLT33_*rhlAB* as template and the primer pair rhlA*B (Table 1) as recommend by the supplier.

### Production of rhamnolipids and HAAs in recombinant *P. putida*

*P. putida* KT2440 was transformed by electroporation as described by Choi et al. (2006). Cells carrying pVLT33-based recombinant plasmids were selected using LB-agar medium or LB liquid medium containing 50 μg/ml kanamycin after incubation at 30 °C.

For the production of rhamnolipids and HAAs, main cultures of 100 ml LB medium in 1 l Erlenmeyer flasks were inoculated to an OD_580_ of 0.05 using overnight cultures and incubated at 30 °C and 150 rpm. The medium was supplemented with 10 g/l glucose, 50 μg/ml kanamycin, and 0.4 mM isopropyl-β-D-thiogalactopyranoside (IPTG) to induce the expression of *rhl*-genes. The cell-free culture supernatant was prepared by centrifugation of the cells for 30 min at 2200×*g* and 4 °C followed by filtration of the supernatant through a cellulose-membrane filter with 0.2 μm pore size (VWR International, Darmstadt, Germany).

### Production of rhamnolipids in *P*. *aeruginosa* mutant strains

For generating *P*. *aeruginosa rhl*-mutant strains, the flanking up- and downstream regions of *rhlA* and *rhlC* were amplified as described above using the primer pairs *rhlA*-up, *rhlA*-down, *rhlC*-up, and *rhlC*-down, respectively (Table 1). PCR products were hydrolyzed with the restriction endonucleases *Pvu*I and *Mlu*I (for upstream regions) and *Mlu*I and *Nco*I (for downstream regions). The two products for each gene were ligated into *Pvu*I and *Nco*I restriction sites of pSUP202 (Simon et al. 1983), creating plasmids pSUP_*rhlA* and pSUP_*rhlC*. Recombinant *E*. *coli* cells containing this vector and its derivatives were selected by adding 10 μg/ml tetracycline to the medium. An Ω-gentamicin-cassette obtained by hydrolysis of pBSL142 (Alexeyev et al. 1995) with *Mlu*I was subsequently cloned into the plasmids pSUP_*rhlA* and pSUP_*rhlC* hydrolyzed with *Mlu*I creating the plasmids pSUP_*rhlA*-Gm and pSUP_*rhlC*-Gm, respectively. Recombinant *E*. *coli* cells containing these plasmids were selected by adding 10 μg/ml gentamicin to the medium. *P*. *aeruginosa* PAO1 was transformed with the gene mutator plasmids by electroporation (Choi et al. 2006) and selected for gentamicin resistance and tetracycline susceptibility indicating homologous recombination events with a double crossing-over. For this purpose, 30 μg/ml gentamicin or 100 μg/ml tetracycline were supplemented to agar-plates and liquid cultures. The *P*. *aeruginosa* mutant strains PAO1Δ*rhlA* and PAO1Δ*rhlC* were cultivated in phosphate-limited protease peptone-glucose-ammonium salt medium (PPGAS), containing 5 g/l glucose, 10 g/l peptone, 0.02 M NH_4_Cl, 0.02 M KCl, 0.12 M Tris-HCl, and 0.0016 M MgSO_4_ adjusted to pH 7.2 (Zhang and Miller, 1992). The main cultures of 10 ml medium in a 100 ml Erlenmeyer flask were inoculated to an OD_580_ of 0.05 from an overnight culture and incubated at 37 °C and 150 rpm. Cell-free culture supernatants were prepared by centrifugation of the cells for 30 min at 2200×*g* and 4 °C followed by filtration of the supernatant through a cellulose-membrane filter with 0.2 μm pore size (VWR International, Darmstadt, Germany).

### Extraction of surface active compounds

For the extraction of rhamnolipids and their precursors HAAs, 4 ml of the cell-free culture supernatants were transferred to a new reaction tube and acidified with 40 μl of phosphoric acid (80% (*v*/*v*)). To these solutions, 6 ml ethyl acetate were added, the samples were mixed on a vortex shaker and centrifuged for 10 min at 2200×*g*. The upper ethyl acetate phases containing rhamnolipids and their precursors were removed. The lower phases were used for second extraction according to the same procedure. The two extracts were combined for further analysis.

For the detection of rhamnolipids using thin layer chromatography (TLC), 1.25 ml and for quantification of rhamnolipids and HAAs via HPLC-UV/Vis analysis 2 ml of the extract were evaporated in the vacuum centrifuge.

### Thin layer chromatography of rhamnolipids

The dried samples for TLC analysis were dissolved in 20 μl ethanol and 10 μl of these solutions were spotted on silica 60 TLC plates (SIL-G, Macherey-Nagel, Düren, Germany). As a positive control, 10 μl of a 0.1% (*w*/*v*) rhamnolipid solution (JBR425, Jeneil Biosurfactant Co., LCC, Saukville, USA) containing mono- and di-rhamnolipids were spotted onto each TLC plate. A mixture of chloroform, methanol, and acetic acid at a ratio of 65:15:2 (*v*/*v*/*v*) was used as mobile phase. To visualize the rhamnolipids, a solution consisting of 0.15 g orcinol-monohydrate, 8.2 ml sulfuric acid, and 42 ml distilled water was sprayed on the TLC plates. The dried plates were incubated at 110 °C for 10 min.

### Quantification of rhamnolipids and HAAs by HPLC-UV/Vis

Crystalline Rha-Rha-C_10_-C_10_ standard was a gift from former Hoechst AG (Frankfurt a. M., Germany). Rha-C_10_-C_10_ and C_10_-C_10_ (HAA) standards for HPLC analysis were prepared and purified as described before (Trummler et al. 2003; Magario et al. 2009). The β-hydroxydecanoic acid (C_10_) standard was obtained from Sigma-Aldrich Chemie GmbH (Steinheim, Germany). For derivatization, triethylamine and 4-bromophenacylbromide were used (Sigma-Aldrich Chemie GmbH) like described by Schenk et al. (1995).

Phenacyl esters of rhamnolipids and β-hydroxydecanoic acids for HPLC analysis were obtained as described before (Schenk et al. 1995) with minor changes. The analysis was performed with a standard HPLC device (Agilent 1100 Series, Agilent Technologies, Waldbronn, Germany) equipped with a Supelcosil® LC-18 Octadecylsilyl (Supelco, Deisenhofen, Germany) reverse phase column (3 mm × 150 mm × 5 μm) at 30 °C. Components of the mobile phase were solution A with 5% (*v*/*v*) methanol and solution B with 95% (*v*/*v*) methanol in ultrapure water, respectively. To achieve separation, a gradient of solution B from 80 to 100% was used according to the following protocol: from *t* = 0 to *t* = 17 min increase of solution B from 80 to 100%, holding 100% solution B up to *t* = 25 min and decrease back to 80% solution B until *t* = 30 min, holding 80% solution B for 5 more min to equilibrate. The injection volume was 10 μl. The flow rate was 0.4 ml/min and analytes were monitored at 254 nm. Retention times were 21.5 (±0.1) min for Rha-Rha-C_10_-C_10_, 22.2 (±0.1) min for Rha-C_10_-C_10_, and 23.3 (±0.1) min for C_10_-C_10_.

### Chemical analysis of rhamnolipids by HPLC-ESI-MS

For the identification of rhamnolipids via HPLC-ESI-MS, the *P. putida* strain carrying pVLT33_*rhlAB* was cultivated in 1 l cultures in 5 l Erlenmeyer flasks and conditions as described before. The rhamnolipids were purified after Déziel et al. (1999) with some modifications. Cells were removed by centrifugation (6740×*g*, 10 °C, 30 min). The supernatant was acidified to pH 3 with 37% HCl and kept at 4 °C and 80 rpm overnight. The precipitated rhamnolipids were recovered by centrifugation (8280×*g*, 10 °C, 45 min) and dissolved in 15 ml acidified water (pH 3, adjusted with 37% HCl). The solution was extracted three times with 15 ml ethyl acetate, the organic phases were collected and evaporated under vacuum. The dried rhamnolipids were dissolved in 15 ml of 0.05 M sodium bicarbonate, acidified to pH 2 with 37% HCl and kept overnight at 4 °C. The rhamnolipids were recovered by centrifugation (4650×*g*, 4 °C, 60 min).

HPLC-MS experiments were carried out on an Agilent 1100 series binary HPLC system (Agilent Technologies, Waldbronn, Germany), combined with a DAD (190–400 nm) and coupled with the triple quadrupole 4000 QTRAP™ mass spectrometer (AB SCIEX, Darmstadt, Germany) equipped with a TurboIon spray source.

Separation was achieved on a ProntoSIL 120-C8-SH (Bischoff Chromatography, Leonberg, Germany) column (2 mm × 150 mm × 3 μm) kept at 20 °C during analysis. The gradient elution was done with deionized water with 0.1% (*v*/*v*) formic acid (solution A) and acetonitrile with 0.1% (*v*/*v*) formic acid (solution B) at a constant flow rate of 300 μl/min in the following manner: start with 60% solution B isocratic for 4 min, from 4 to 24 min a linear increase from 60% solution B to 90% solution B, followed by a second isocratic step (90% solution B for 10 min). The return to 60% solution B was performed in 1 min and 10 min; isocratic (60% solution B) was used for the re-equilibration. The injection volume was 20 μl.

The MS was used in the negative EMS mode scanning from 200 to 1000 Da. The parameters used were optimized first performing a flow injection analysis (FIA) with a standard and led to the following parameter settings: IS −4500 V, declustering potential (DP) −100 V, curtain gas (N_2_) 10 arbitrary units (au), source temperature 500 °C, nebulizer gas (N_2_) 50 au, and heater gas (N_2_) 20 au. Collision energy (CE) and Q3-entry barrier were set to −5 V and 8 V, respectively, to minimize fragmentation entering the LIT in the full scan mode.

For structural elucidation, MS/MS experiments were performed in negative enhanced product ion (EPI) scan mode. CE in the range between 30 and 70 V were used.

## Results

### Biosynthesis of HAAs and mono-rhamnolipids in recombinant *P*. *putida*

Typical mono- and di-rhamnolipids contain two 3-hydroxyfatty acid chains; however, rhamnolipid congeners with only one 3-hydroxyfatty acid chain were described, too (Syldatk et al. 1985b; Déziel et al. 1999). Two different routes for the biosynthesis of these mono-rhamno-mono-lipids were suggested. The first is of anabolic character and proposes a RhlB catalyzed transfer of a single ACP-activated 3-hydroxyfatty acid to a dTDP-L-rhamnose molecule. The second catabolic approach involves a hypothetic degradation step of common mono-rhamno-di-lipids by an unknown hydrolytic enzyme.

To investigate the biosynthetic pathway for mono-rhamno-mono-lipids, we used *P*. *putida* KT2440 for which we previously reported multiple advantages for heterologous rhamnolipid production in comparison with *P*. *aeruginosa* (Wittgens et al. 2011; Tiso et al. 2016). The fact that this strain does not produce rhamnolipids itself simplifies the analysis of different rhamnolipid species after the expression of *P*. *aeruginosa* PAO1 genes involved in rhamnolipid biosynthesis.

**Fig. 2:**
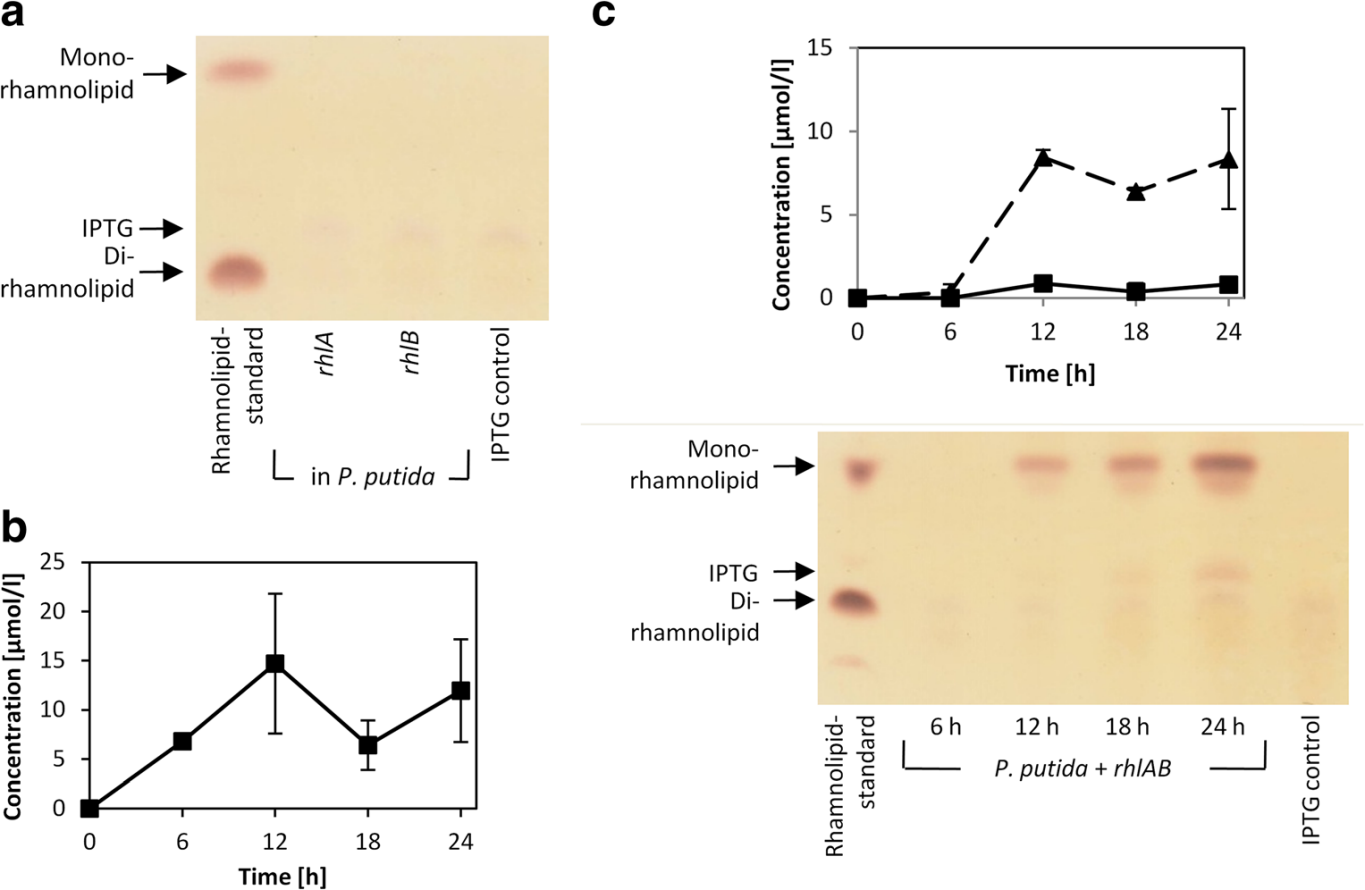
Rhamnolipids and HAAs produced by recombinant *P. putida*. **a** Thin layer chromatography (TLC) of extracts from single *rhlA* or *rhlB* expression shows no detectable amounts of rhamnolipids after 24 h (HPLC results not shown). **b** HPLC analysis of HAAs reveals their production in a *rhlA* expressing *P. putida* strain. **c** HPLC analysis of HAAs (*squares*) and mono-rhamnolipids (*triangles*) and TLC of *P. putida* cultures carrying *rhlAB* operon. Rhamnolipids are visible as *brown bands* on TLC plates as in the rhamnolipid-standard. Samples extracted from *P. putida* cultures show an additional *violet spot* descending from IPTG as in extracts of IPTG containing LB media (IPTG control). Samples were taken every 6 h for a period of 24 h from three independent cultures

For this purpose, the pVLT33_*rhlA*, pVLT33_*rhlB*, and pVLT33_*rhlAB* expression vectors, respectively carrying the single *rhlA* gene, *rhlB* gene, or the *rhlAB* operon, were used (Table 1). The heterologous expression of the single *rhlA* or *rhlB* genes in *P*. *putida* KT2440 did not yield detectable amounts of rhamnolipids after 24 h (Fig. 2a). However, as expected, the bacteria expressing *rhlA* secreted up to 12.0 μmol/l HAAs within 24 h confirming known catalytic activity of produced RhlA as HAA synthase (Fig. 2b). Furthermore, the production of 8.5 μmol/l mono-rhamnolipids was observed by expressing the *rhlAB* operon in *P*. *putida* for 24 h confirming that RhlB was expressed in its enzymatically active form as well (Fig. 2c).

The chemical analysis of the secreted mono-rhamnolipids produced by recombinant *P*. *putida* expressing *rhlAB* by HPLC-ESI-MS revealed six different mono-rhamnolipid congeners containing two 3-hydroxyfatty acids with chain length between C_8_-C_10_ and C_12_-C_12_ and a predominant C_10_-C_10_ species, which correlates with the composition of rhamnolipids known from *P*. *aeruginosa* (Abdel-Mawgoud et al. 2010). Beside the predominant mono-rhamno-di-lipids, also two mono-rhamno-mono-lipid congeners (Rha-C_8_ and Rha-C_10_) were detectable.

### RhlA and RhlB function independently from each other

Surprisingly, *P*. *putida* expressing *rhlAB* produced also mono-rhamno-mono-lipids while these compounds were not detected in the supernatant of *P*. *putida* expressing only the *rhlB* gene, although the function of RhlB for transfer of a single 3-hydroxyacyl-ACP to a dTDP-L-rhamnose molecule has been suggested (Soberón-Chávez et al. 2005). In the literature RhlA and RhlB are described as two subunits of rhamnosyltransferase I forming a heterodimer to build the active enzyme complex (www.ncbi.nlm.nih.gov; Ochsner et al. 1994a; Winsor et al. 2016). We examined the role of this RhlAB complex for the biosynthesis of rhamnolipids and could show that RhlA can exert its function independently of RhlB (Fig. 2b). In addition, our results reveal that RhlB was active after expression of the *rhlAB* operon but not as single protein in *P*. *putida* (Fig. 2c). The fact that RhlB was active in the presence of RhlA but not when *rhlA* was not co-expressed opened the question if the function of RhlA is to stabilize RhlB through protein-protein interactions or if RhlA indirectly affect the function of RhlB through the supply of HAAs as substrate for RhlB.

**Fig. 3:**
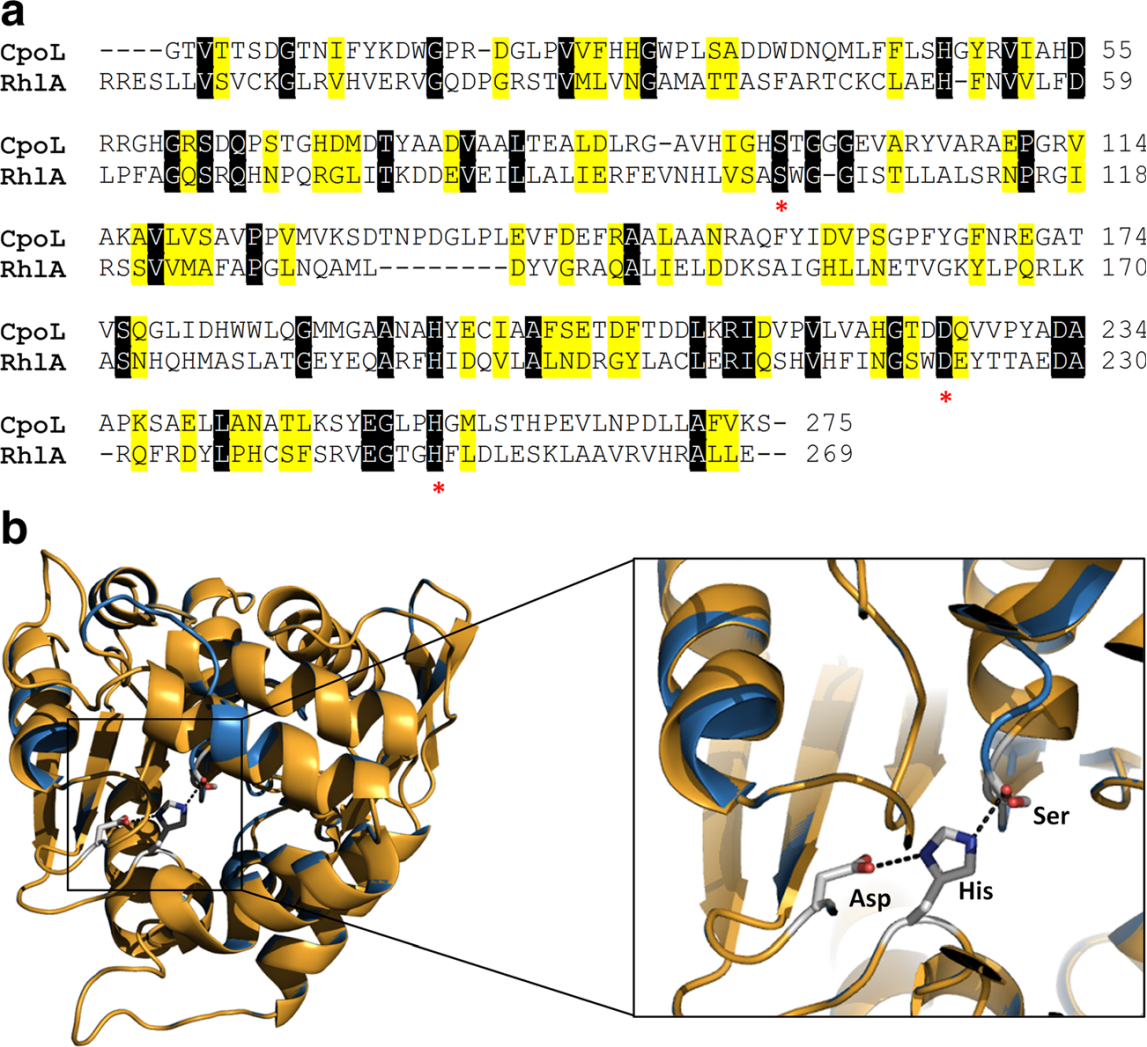
*Identification of the catalytic triade of RhlA*. **a** The three-dimensional structure of RhlA from *P. aeruginosa* was modeled using the chloroperoxidase L (CpoL; PDB code: 1A88) from *Streptomyces lividans* as template. Despite low sequence identity (14%), the catalytic triad Ser, Asp, and His (indicated by an *asterisk* underneath the sequences) are strongly conserved among these two proteins. Sequences identical and similar were shaded in *black* and *yellow*, respectively. **b** Structural superimposition of CpoL (*brown*) and RhlA (*blue*) shows a high conservation of secondary structure elements. The catalytic triad of CpoL (Ser96, His255 and Asp226) and the putative catalytic triad of RhlA (Ser102, His251, and Asp223) are structurally strongly conserved. *Dashed lines* indicate catalytically important interactions of the active site residues

To investigate the hypothesis that RhlB requires RhlA for its stabilization, a catalytically inactive variant of RhlA was constructed and expressed together with RhlB. The active site of RhlA was identified by structural homology modeling using the *S*. *lividans* chloroperoxidase L, a protein of the α/β-hydrolase superfamily, as a template (Fig. 3a). The residues Ser98, Asp228, and His257 described as the catalytic triad residues of chloroperoxidase L (Hofmann et al. 1998) were structurally conserved with Ser102, Asp223, and His251 of RhlA, respectively (Fig. 3b). Thus, we substituted the predicted catalytic serine of RhlA at position 102 by alanine. Serine to alanine mutations are generally used to inactivate serine hydrolases because alanine cannot take over the nucleophile function of serine as a substitute, but maintains the structural integrity of the protein (Kovačić et al. 2013).

Expression of the rhlA*B operon, encoding the inactive RhlA* variant and wild-type RhlB, in *P*. *putida* did not result in the production neither of HAAs nor of any mono-rhamnolipid species (data not shown). This confirmed Ser102 being the active site residue of RhlA as no HAAs were synthesized. Furthermore, this result is in conflict with the hypothesis of a functional RhlAB complex, as in this strain inactive RhlA* can form a complex with RhlB; however, RhlB is unable to produce mono-rhamnolipids. Conclusively, RhlB function is dependent on supply of precursors by RhlA rather than on interaction with RhlA.

### Synthesis of mono-rhamnolipids by RhlB after uptake of extracellular HAAs as precursors

**Fig. 4:**
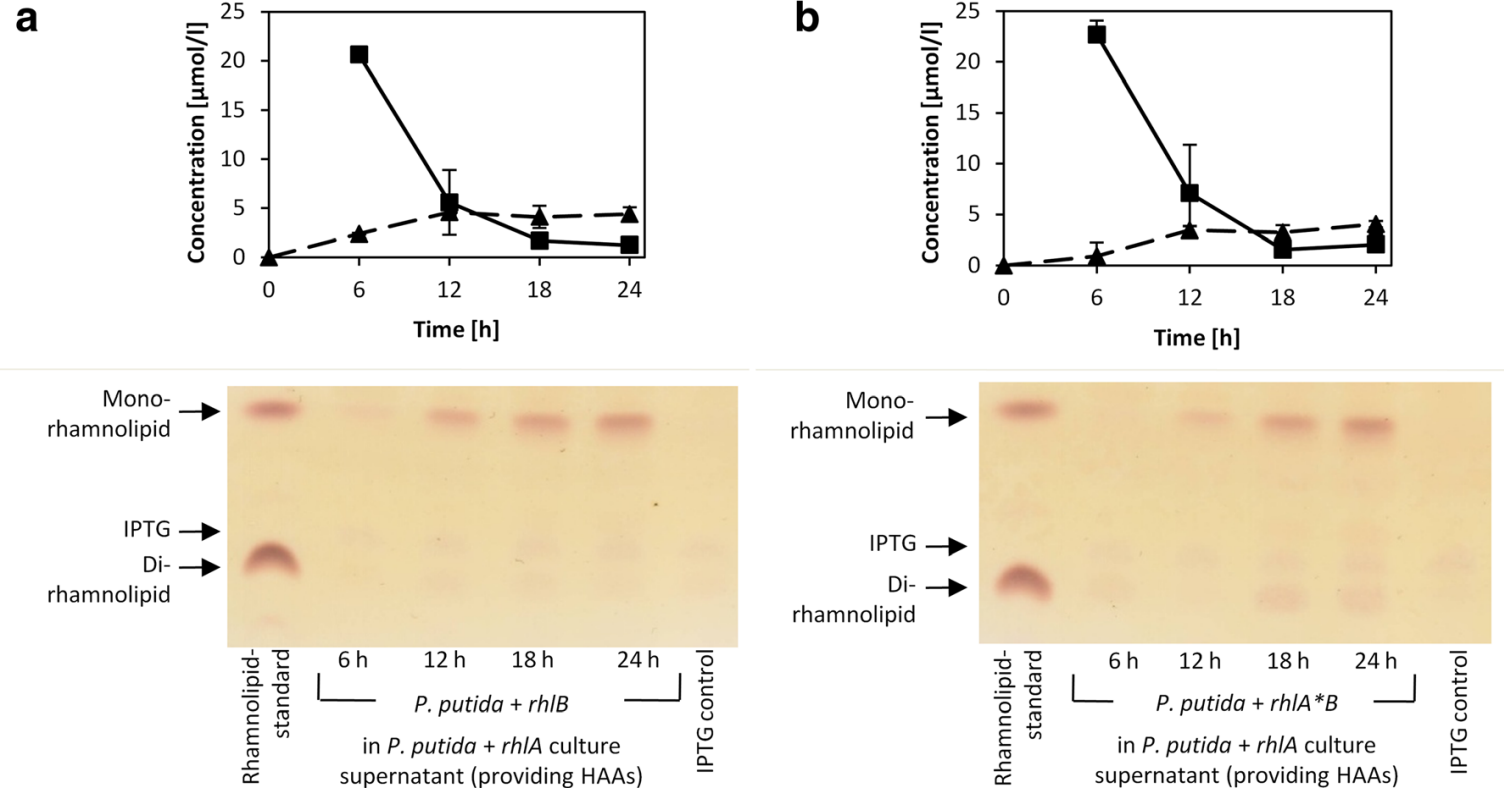
*Production of mono-rhamnolipids by recombinant P. putida in HAA containing conditioned medium*. *P. putida* strains expressing single *rhlB* (**a**) or the rhlA*B operon (**b**), containing inactive RhlA, were cultivated in HAA containing conditioned medium, obtained from a *rhlA* expressing *P. putida* strain. Extracts were analyzed via HPLC revealing HAAs (*squares*) and mono-rhamnolipids (*triangles*) and thin layer chromatography. Rhamnolipids are visible as brown bands on TLC plates as in the rhamnolipid-standard. Samples extracted from *P. putida* cultures show an additional violet spot descending from IPTG as in extracts of IPTG containing LB media (IPTG control). Samples were taken every 6 h for a period of 24 h from three independent cultures

To verify that RhlB catalyzed biosynthesis of mono-rhamnolipids is dependent on the supply of HAA precursors synthesized by RhlA, experiments with conditioned media containing HAAs were performed. The conditioned media were obtained by mixing cell-free supernatant of *P*. *putida* cultures expressing the *rhlA* gene responsible for HAA synthesis with the same volume of fresh LB medium. HPLC results revealed that *P*. *putida* expressing *rhlB* and *P*. *putida* expressing the inactive rhlA*B operon cultivated in conditioned medium produced comparable amounts (4.5 μmol/l for *rhlB* and 4.0 μmol/l for rhlA*B) of extracellular mono-rhamnolipids within 24 h of incubation (Fig. 4a, b). Time course experiments showed that at the same time, the concentration of HAAs decreased from 20.5 to 1.5 μmol/l and from 22.5 to 2.0 μmol/l for strains expressing *rhlB* and rhlA*B, respectively (Fig. 4a, b). These results point out that HAAs synthesized by *P*. *putida* and provided in the form of the conditioned medium serve as the substrate for RhlB. Moreover, the expression of catalytically inactive RhlA does not significantly affect the efficiency of RhlB to synthesize mono-rhamnolipids. Thus, we conclude that RhlB may in fact catalyze the formation of the β-glycosidic bond between HAA and dTDP-L-rhamnose independently on the presence of RhlA but is dependent on RhlA through the supply of HAAs.

### Synthesis of di-rhamnolipids by RhlC using extracellular mono-rhamnolipids as precursors

Our results demonstrate for the first time that *P*. *putida* can take up exogenous HAAs for the synthesis of mono-rhamnolipids. Therefore, we tested if mono-rhamnolipids can be imported by *P*. *putida* cells as well and if they flow into the di-rhamnolipid biosynthesis pathway, which relies on RhlC. In addition, we investigated the function of PA1131, which is organized in a bicistronic operon together with *rhlC* and has been supposed to play a role in rhamnolipid secretion (Rahim et al. 2001), but experimental evidences were currently missing.

**Fig. 5:**
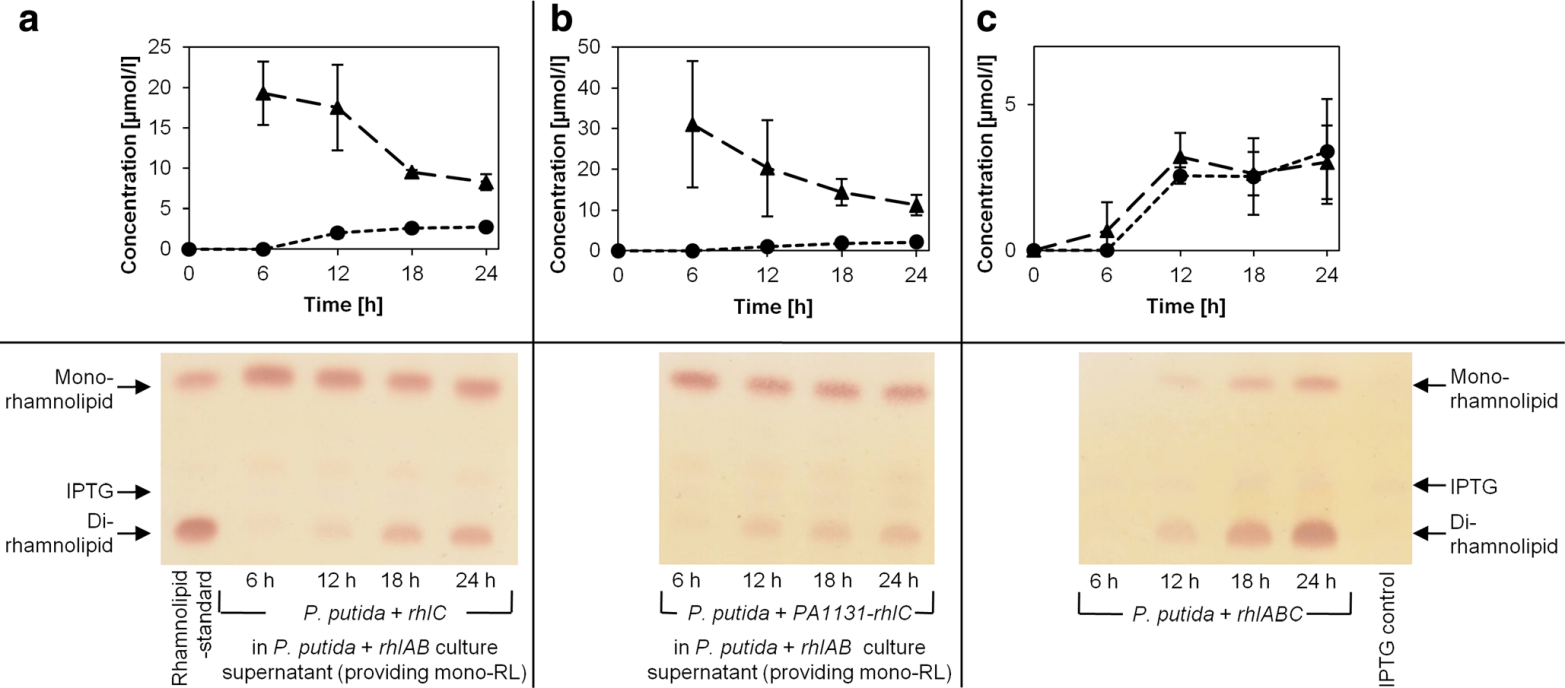
*Production of di-rhamnolipids by recombinant P. putida in mono-rhamnolipid containing conditioned medium*. *P. putida* strains expressing single *rhlC* (**a**) or the *PA1131*-*rhlC* operon (**b**) were cultivated in mono-rhamnolipid containing conditioned medium, obtained from a *rhlAB* expressing *P. putida* strain. **c** For comparison, *P. putida* expressing the biosynthetic *rhlABC* operon cultivated in fresh LB media. Extracts were analyzed via HPLC revealing HAAs (*squares*), mono-rhamnolipids (*triangles*), and di-rhamnolipids (*circles*) and thin layer chromatography. Rhamnolipids are visible as *brown bands* on TLC plates as in the rhamnolipid-standard. Samples extracted from *P. putida* cultures show an additional *violet spot* descending from IPTG as in extracts of IPTG containing LB media (IPTG control). Samples were taken every 6 h for a period of 24 h from three independent cultures

For this purpose, *P*. *putida* carrying pVLT33_*rhlC* or pVLT33_*PA1131*-*rhlC* (Table 1) were cultivated in conditioned medium containing mono-rhamnolipids. The conditioned medium was obtained by mixing cell-free supernatants of *P*. *putida* expressing the *rhlAB* operon, which produces mono-rhamnolipids, with the same volume of fresh LB medium. HPLC analysis showed that the amount of the mono-rhamnolipids in the culture medium of *P*. *putida* expressing *rhlC* decreased from 19.5 to 8.5 μmol/l, while the amount of di-rhamnolipids reached 2.5 μmol/l during 24 h of cultivation (Fig. 5a). The *P*. *putida* strain expressing the *PA1131*-*rhlC* operon was able to uptake mono-rhamnolipids and synthesized di-rhamnolipids with similar efficiency as the *P*. *putida* expressing only *rhlC* (Fig. 5b). However, cultivation of *P*. *putida* expressing a biosynthetic *rhlABC* operon in fresh LB medium resulted in a mixture consisting of 3.0 μmol/l mono-rhamnolipids and 3.5 μmol/l di-rhamnolipids (Fig. 5c).

Our results demonstrate that *P*. *putida* indeed can take up mono-rhamnolipids from the medium and use them as a precursor for the subsequent synthesis of di-rhamnolipids. Moreover, under the experimental conditions tested here, the protein of unknown function PA1131 has neither quantitative nor qualitative influence on rhamnolipid biosynthesis and does not appear to be involved in excretion of rhamnolipids.

### *P*. *aeruginosa* can also import extracellular mono-rhamnolipids as precursors for RhlC-dependent di-rhamnolipid synthesis

The discovery of rhamnolipid precursor uptake by *P*. *putida* motivated us to examine if similar processes can be observed for the native rhamnolipid producer *P*. *aeruginosa*.

**Fig. 6:**
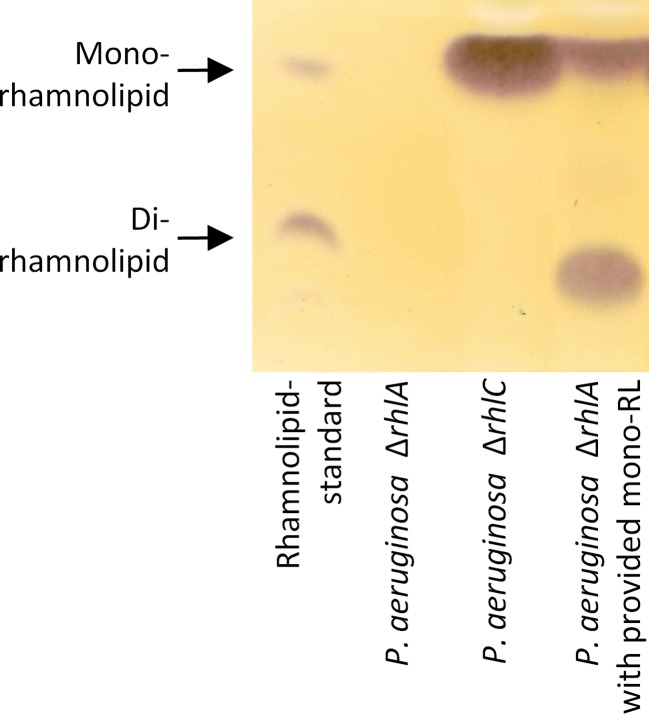
*Production of rhamnolipids by P. aeruginosa rhl-mutant strains*. Thin layer chromatography was performed to analyze rhamnolipid biosynthesis by *P. aeruginosa* Δ*rhlA* and *P. aeruginosa* Δ*rhlC* cultivated in PPGAS medium. In addition, *P. aeruginosa* Δ*rhlA* was cultivated in mono-rhamnolipid containing conditioned medium, obtained from a *P. aeruginosa* Δ*rhlC* culture. Samples were taken after 24 h. Rhamnolipids are visible as *brown bands* on TLC plates as in the rhamnolipid-standard

Cultivating *P*. *aeruginosa* PAO1Δ*rhlC*, which lacks rhamnosyltransferase II RhlC, but still expresses *rhlAB*, in PPGAS medium leads to the production of mono-rhamnolipids (Fig. 6). The obtained mono-rhamnolipid containing conditioned medium mixed with an equal volume of fresh PPGAS was used for the cultivation of *P*. *aeruginosa* PAO1Δ*rhlA*. Our results demonstrate that this *rhlA* mutant strain, which still expresses functional rhamnosyltransferase II RhlC, transforms the externally provided mono-rhamnolipids into di-rhamnolipids after resumption (Fig. 6). However, based on *rhlAB* knock-out, this mutant strain is not able to synthesize any kind of rhamnolipids by itself cultivated in PPGAS medium (Fig. 6). In this experiment, the di-rhamnolipids of the standard showed an untypical mobility on the TLC plate (Fig. 6) in comparison to the di-rhamnolipids of the sample. This commercial rhamnolipid-standard was obtained with only minor information about origin, purification procedure, or ingredients except the contained rhamnolipid species. The different ingredients possibly influence the mobility of di-rhamnolipids under certain conditions. However, the *R*_*f*_ value of di-rhamnolipids was identical in all sample lanes during this work at 0.27 representing di-rhamnolipids with a predominant Rha-Rha-C_10_-C_10_ species, which was verified by HPLC-ESI-MS analysis.

## Discussion

The biosynthesis of mono-rhamnolipids requires the two enzymes RhlA and RhlB, which are encoded by genes organized in the bicistronic *rhlAB* operon. These enzymes according to presently accepted hypotheses fulfill their functions in the form of a heterodimeric rhamnosyltransferase I enzyme complex (Ochsner et al. 1994a). Hence, in the Pseudomonas database (Winsor et al. 2016), RhlA and RhlB are respectively annotated as chain A and chain B of rhamnosyltransferase I. We could show that in *P*. *putida* RhlA with its 3-hydroxyacyl-ACP:3-hydroxyacyl-ACP O-3-hydroxyacyltransferase activity is responsible for the production of the rhamnolipid precursor molecule HAA and is not dependent on the expression of *rhlB*, what is also known from experiments with *E*. *coli* as a model host organism (Lépine et al. 2002; Déziel et al. 2003; Zhu and Rock 2008). However, if the hypotheses were true that RhlB could catalyze the acylation of dTDP-L-rhamnose with one ACP activated 3-hydroxyfatty acid (Soberón-Chávez et al. 2005), the production of mono-rhamno-mono-lipids were expected at least, but single expression of the *rhlB* gene in *P*. *putida* did not result in any rhamnolipid production. The same result was achieved when the mutagenized rhlA*B operon containing an inactive RhlA was expressed. RhlA inactivation was done in order to separate the postulated dual functionality of this protein as an enzyme and as a stabilizing function for RhlB in the postulated complex. However, since extracellularly provided HAAs are a substrate for rhamnolipid biosynthesis in *P*. *putida* expressing the single *rhlB* gene or the rhlA*B operon, we conclude that RhlB is active as a single enzyme and is most likely not a subunit of the rhamnosyltransferase I enzyme complex. Thus, RhlB fulfills the role as rhamnosyltransferase I independent of the assistance of RhlA for its stabilization, but strongly requires the RhlA product HAA as substrates for its own activity.

Given that RhlB cannot use single 3-hydroxyfatty acids for the synthesis of mono-rhamno-mono-lipids, these relatively rare rhamnolipid species containing only one fatty acid chain then have to descend from hydrolysis of typical and more abundant rhamnolipid species containing the normal dimer of two 3-hydroxyfatty acids. Since mono-rhamno-mono-lipids also occur in *P*. *putida*, it is reasonable to suspect that *P*. *putida* as well as *P*. *aeruginosa* contain at least one enzymatic activity, which is able to hydrolyze the ester bond between the two 3-hydroxyfatty acids of rhamnolipids. Taking into account the typical chain lengths of fatty acids of 8 to 14 carbon atoms, the most probable enzymes for this activity can be expected to belong to the esterase or lipase family of hydrolases. *P*. *aeruginosa*, for which these rhamnolipid species were first described (Syldatk et al. 1985a; Déziel et al. 1999), produces several lipolytic enzymes (Wilhelm et al. 1999; Leščić Ašler et al. 2010; Funken et al. 2011; Kovačić et al. 2013; Kovacic et al. 2016). However, 80 putative are predicted for *P*. *aeruginosa* (Jaeger and Kovacic 2014) and about half as many for *P*. *putida* (Nelson et al. 2002; Winsor et al. 2016). The identification of the individual enzymes responsible for modification of rhamnolipids by processing of fatty acids will be the aim of a further study and may provide a tool to enlarge the product range for designer rhamnolipids containing hydrophobic moieties composed of uneven numbers of fatty acids. Since rhamnolipid species containing, e.g., only one fatty acid feature an entirely different chemical proportion between the hydrophilic and hydrophobic molecule domain in comparison to typical rhamnolipids, they are considerably different and thus highly interesting for various novel applications, because their characteristics strongly affect the physicochemical properties of surfactants (Winsor 1954; Salager et al. 1979; Acosta et al. 2008).

Furthermore, extracellularly provided mono-rhamnolipids were converted to di-rhamnolipids by RhlC in recombinant *P*. *putida*. This reaction could be observed upon expression of *rhlC* as a single gene and also by expression of the *PA1131-rhlC* operon. The gene *PA1131* was predicted to probably encode a transporter of the major facilitator superfamily (MFS) and the genetically organization in an operon together with *rhlC* consequently led to the hypothesis that the resulting protein might be involved in rhamnolipid production and/or secretion (Rahim et al. 2001). However, the additional expression of this gene had no influence on the rhamnolipid production or secretion in the recombinant *P*. *putida* strains. In addition, the genome of *P. putida* (Nelson et al. 2002) does not contain any gene for a homologous protein that could substitute or complement the suggested role as a membrane transporter, suggesting that PA1131 has probably a role different from rhamnolipid transport and thus may be, if at all, more indirectly linked to rhamnolipid production.

Furthermore, this is the first report that exogenously added HAAs and mono-rhamnolipids can cross both membranes of the original host *P*. *aeruginosa* and of the heterologous host *P*. *putida* to reach the cytoplasm, where all the proteins responsible for rhamnolipid biosynthesis are located (Rahim et al. 2001; Winsor et al. 2016). This suggests that beside the yet unknown exporter for HAAs and rhamnolipids, there probably may exist an importer system for the resumption of these compounds. However, the mechanism behind this retrograde transport is currently completely unknown as are candidate proteins for this functionality. A simple diffusion of the tensioactive molecules seems rather unlikely because their transport by diffusion would require close and direct interactions of HAA and rhamnolipids with the lipid bilayers of the cell membranes. One function of rhamnolipids is to emulsify hydrophobic substrates like long-chain hydrocarbons and oil in the extracellular milieu (Zhang and Miller 1992; Patel and Desai 1997) or to bind to the cell surface and to modify its overall hydrophobicity, which in turn leads to the enhanced uptake of these substrates into the cell (Zhang and Miller 1994; Noordman and Janssen 2002). In this process, below its critical micelle concentration (CMC), the hydrophilic moiety of rhamnolipids interacts with O-antigen component of lipopolysaccharides (LPS) in the outer membrane of Gram-negative bacteria to increase its hydrophobicity (Zhong et al. 2008). Above the CMC, rhamnolipids remove LPS from the outer membrane, which still leads to an increase in hydrophobicity due to the resulting lack of polar sugar residues on the cell surface (Al-Tahhan et al. 2000; Sotirova et al. 2009). It is still speculative, if rhamnolipids only permit the interaction between cell surface and hydrophobic substrates or if the cells can absorb larger adducts of substrate molecules or particles emulsified by micelles of rhamnolipids or HAAs, which also show tensioactive properties (Lépine et al. 2002). Speculative, but possibly rhamnolipid producing organisms use a cycle of HAA and rhamnolipid secretion, emulsification of hydrophobic compounds, and their reabsorption into the cell to metabolize the encased substrate and to recycle the surfactants.

Surprisingly, the decreasing amounts of HAAs and mono-rhamnolipids presented in the experiments do not quantitatively reflect the increasing amount of mono- and di-rhamnolipids, respectively. This difference indicates that *P. putida* can actively degrade the surfactants and is probably able to metabolize the breakdown products of these molecules. The hypothetical lipolytic enzyme responsible for the hydrolysis of rhamnolipids and probably also free HAAs into free fatty acids and mono-rhamno-mono-lipids may be also the catalyst in this process. For complete degradation of the latter, a second enzyme similar to the naringinase from *Aspergillus niger* (Trummler et al. 2003) would be essentially required to hydrolyze the β-glycosidic bond and release the rhamnose moiety. The identification of these enzymes will be one topic of further investigations to step forward to understand the complete biochemistry of rhamnolipid production and metabolism, to create a toolbox of enzymatic activities on HAAs and rhamnolipids and—in perspective—to open novel strategies based on these enzymes for the production of tailor made rhamnolipid molecules with specific properties for biomedical and conventional applications in biotechnology and industry.

## References

[CR1] Abalos A, Pinazo A, Infante MR, Casals M, Garcia F, Manresa A (2001). Physicochemical and antimicrobial properties of new rhamnolipids produced by *Pseudomonas aeruginosa* AT10 from soybean oil refinery wastes. Langmuir.

[CR2] Abdel-Mawgoud AM, Lépine F, Déziel E (2010). Rhamnolipids: diversity of structures, microbial origins and roles. Appl Microbiol Biotechnol.

[CR3] Acosta EJ, Yuan JS, Bhakta AS (2008). The characteristic curvature of ionic surfactants. J Surfactant Deterg.

[CR4] Alexeyev MF, Shokolenko IN, Croughan TP (1995). Improved antibiotic-resistance gene cassettes and omega elements for *Escherichia coli* vector construction and in vitro deletion/insertion mutagenesis. Gene.

[CR5] Alhede M, Bjarnsholt T, Jensen PØ, Phipps RK, Moser C, Christophersen L, Christensen LD, van Gennip M, Parsek M, Høiby N, Rasmussen TB, Givskov M (2009). *Pseudomonas aeruginosa* recognizes and responds aggressively to the presence of polymorphonuclear leukocytes. Microbiology.

[CR6] Al-Tahhan RA, Sandrin TR, Bodour AA, Maier RM (2000). Rhamnolipid-induced removal of lipopolysaccharide from *Pseudomonas aeruginosa*: effect on cell surface properties and interaction with hydrophobic substrates. Appl Environ Microbiol.

[CR7] Andrä J, Rademann J, Howe J, Koch MHJ, Heine H, Zähringer U, Brandenburg K (2006). Endotoxin-like properties of a rhamnolipid exotoxin from *Burkholderia* (*Pseudomonas*) *plantarii*: immune cell stimulation and biophysical characterization. Biol Chem.

[CR8] Banat I, Franzetti A, Gandolfi I, Bestetti G, Martinotti M, Fracchia L, Smyth T, Marchant R (2010). Microbial biosurfactants production, applications and future potential. Appl Microbiol Biotechnol.

[CR9] Behrens B, Engelen J, Tiso T, Blank LM, Hayen H (2016). Characterization of rhamnolipids by liquid chromatography/mass spectrometry after solid-phase extraction. Anal Bioanal Chem.

[CR10] Beuker J, Steier A, Wittgens A, Rosenau F, Henkel M, Hausmann R (2016). Integrated foam fractionation for heterologous rhamnolipid production with recombinant *Pseudomonas putida* in a bioreactor. AMB Express.

[CR11] Choi KH, Kumar A, Schweizer HP (2006). A 10-min method for preparation of highly electrocompetent *Pseudomonas aeruginosa* cells: application for DNA fragment transfer between chromosomes and plasmid transformation. J Microbial Methods.

[CR12] Davey ME, Caiazza NC, O'Toole GA (2003). Rhamnolipid surfactant production affects biofilm architecture in *Pseudomonas aeruginosa* PAO1. J Bacteriol.

[CR13] de Lorenzo V, Eltis L, Kessler B, Timmis KN (1993). Analysis of *Pseudomonas* gene products using *lacI*^q^/*Ptrp-lac* plasmids and transposons that confer conditional phenotypes. Gene.

[CR14] DeLano WL (2002). The PyMOL molecular graphics system.

[CR15] Déziel E, Lépine F, Dennie D, Boismenu D, Mamer OA, Villemur R (1999). Liquid chromatography/mass spectrometry analysis of mixtures of rhamnolipids produced by *Pseudomonas aeruginosa* strain 57RP grown on mannitol or naphthalene. Biochim Biophys Acta.

[CR16] Déziel E, Lépine F, Milot S, Villemur R (2003). *rhlA* is required for the production of a novel biosurfactant promoting swarming motility in *Pseudomonas aeruginosa*: 3-(3-hydroxyalkanoyloxy)alkanoic acids (HAAs), the precursors of rhamnolipids. Microbiology.

[CR17] Dubeau D, Déziel E, Woods DE, Lépine F (2009). *Burkholderia thailandensis* harbors two identical *rhl* gene clusters responsible for the biosynthesis of rhamnolipids. BMC Microbiol.

[CR18] Funken H, Knapp A, Vasil ML, Wilhelm S, Jaeger KE, Rosenau F (2011). The lipase LipA (PA2862) but not LipC (PA4813) from *Pseudomonas aeruginosa* influences regulation of pyoverdine production and expression of the sigma factor PvdS. J Bacteriol.

[CR19] Funston SJ, Tsaousi K, Rudden M, Smyth TJ, Stevenson PS, Marchant R, Banat IM (2016). Characterising rhamnolipid production in *Burkholderia thailandensis* E264, a non-pathogenic producer. Appl Microbiol Biotechnol.

[CR20] Giani C, Wullbrandt D, Rothert R, Meiwes J (1997) *Pseudomonas aeruginosa* and its use in a process for the biotechnological preparation of L-rhamnose. US005658793A. Hoechst AG, Frankfurt a. M.

[CR21] Grant SGN, Jessee J, Bloom FR, Hanahan D (1990). Differential plasmid rescue from transgenic mouse DNAs into *Escherichia coli* methylation-restriction mutants. Proc Natl Acad Sci U S A.

[CR22] Hanahan D (1983). Studies on transformation of *Escherichia coli* with plasmids. J Mol Biol.

[CR23] Hancock RE, Carey AM (1979). Outer membrane of *Pseudomonas aeruginosa*: heat- and 2-mercaptoethanol-modifiable proteins. J Bacteriol.

[CR24] Häußler S, Nimtz M, Domke T, Wray V, Steinmetz I (1998). Purification and characterization of a cytotoxic exolipid of *Burkholderia pseudomallei*. Infect Immun.

[CR25] Hauser G, Karnovsky ML (1957). Rhamnose and rhamnolipide biosynthesis by *Pseudomonas aeruginosa*. J Biol Chem.

[CR26] Henkel M, Schmidberger A, Kühnert C, Beuker J, Bernard T, Schwartz T, Syldatk C, Hausmann R (2013). Kinetic modeling of the time course of N-butyryl-homoserine lactone concentration during batch cultivations of *Pseudomonas aeruginosa* PAO1. Appl Microbiol Biotechnol.

[CR27] Hofmann B, Tölzer S, Pelletier I, Altenbuchner J, van Pée KH, Hecht HJ (1998). Structural investigation of the cofactor-free chloroperoxidases. J Mol Biol.

[CR28] Jaeger KE, Kovacic F (2014). Determination of lipolytic enzyme activities. Methods Mol Biol.

[CR29] Jarvis FG, Johnson MJ (1949). A glycolipide produced by *Pseudomonas aeruginosa*. J Am Chem Society.

[CR30] Johann S, Seiler TB, Tiso T, Bluhm K, Blank LM, Hollert H (2016). Mechanism-specific and whole-organism ecotoxicity of mono-rhamnolipids. Sci Total Environ.

[CR31] Kelley LA, Sternberg MJ (2009). Protein structure prediction on the web: a case study using the Phyre server. Nat Protoc.

[CR32] Köhler T, Curty LK, Barja F, van Delden C, Pechère JC (2000). Swarming of *Pseudomonas aeruginosa* is dependent on cell-to-cell signaling and requires flagella and pili. J Bacteriol.

[CR33] Kovacic F, Bleffert F, Caliskan M, Wilhelm S, Granzin J, Batra-Safferling R, Jaeger KE (2016). A membrane-bound esterase PA2949 from *Pseudomonas aeruginosa* is expressed and purified from *Escherichia coli*. FEBS Open Bio.

[CR34] Kovačić F, Granzin J, Wilhelm S, Kojić-Prodić B, Batra-Safferling R, Jaeger KE (2013). Structural and functional characterisation of TesA—a novel lysophospholipase A from *Pseudomonas aeruginosa*. PLoS One.

[CR35] Kownatzki R, Tümmler B, Döring G (1987). Rhamnolipid of *Pseudomonas aeruginosa* in sputum of cystic fibrosis patients. Lancet.

[CR36] Lang S, Wullbrandt D (1999). Rhamnose lipids—biosynthesis, microbial production and application potential. Appl Microbiol Biotechnol.

[CR37] Lépine F, Déziel E, Milot S, Villemur R (2002). Liquid chromatographic/mass spectrometric detection of the 3-(3-hydroxyalkanoyloxy) alkanoic acid precursors of rhamnolipids in *Pseudomonas aeruginosa* cultures. J Mass Spectrom.

[CR38] Leščić Ašler I, Ivić N, Kovačić F, Schell S, Knorr J, Krauss U, Wilhelm S, Kojić-Prodić B, Jaeger KE (2010). Probing enzyme promiscuity of SGNH hydrolases. Chembiochem.

[CR39] Magario I, Vielhauer O, Neumann A, Hausmann R, Syldatk C (2009). Kinetic analysis and modeling of the liquid-liquid conversion of emulsified di-rhamnolipids by Naringinase from *Penicillium decumbens*. Biotechnol Bioeng.

[CR40] Maier RM, Soberón-Chávez G (2000). *Pseudomonas aeruginosa* rhamnolipids: biosynthesis and potential applications. Appl Microbiol Biotechnol.

[CR41] Manso Pajarron A, de Koster CG, Heerma W, Schmidt M, Haverkamp J (1993). Structure identification of natural rhamnolipid mixtures by fast atom bombardment tandem mass spectrometry. Glycoconj J.

[CR42] Maslin P, Maier RM (2000). Rhamnolipid-enhanced mineralization of phenanthrene in organic-metal co-contaminated soils. Bioremed J.

[CR43] McClure CD, Schiller NL (1996). Inhibition of macrophage phagocytosis by *Pseudomonas aeruginosa* rhamnolipids in vitro and in vivo. Curr Microbiol.

[CR44] Müller MM, Hausmann R (2011). Regulatory and metabolic network of rhamnolipid biosynthesis: traditional and advanced engineering towards biotechnological production. Appl Microbiol Biotechnol.

[CR45] Müller MM, Hörmann B, Kugel M, Syldatk C, Hausmann R (2011). Evaluation of rhamnolipid production capacity of *Pseudomonas aeruginosa* PAO1 in comparison to the rhamnolipid over-producer strains DSM 7108 and DSM 2874. Appl Microbiol Biotechnol.

[CR46] Nelson KE, Weinel C, Paulsen IT, Dodson RJ, Hilbert H, Martins dos Santos VAP, Fouts DE, Gill SR, Pop M, Holmes M, Brinkac L, Beanan M, DeBoy RT, Daugherty S, Kolonay J, Madupu R, Nelson W, White O, Peterson J, Khouri H, Hance I, Chris Lee P, Holtzapple E, Scanlan D, Tran K, Moazzez A, Utterback T, Rizzo M, Lee K, Kosack D, Moestl D, Wedler H, Lauber J, Stjepandic D, Hoheisel J, Straetz M, Heim S, Kiewitz C, Eisen JA, Timmis KN, Düsterhöft A, Tümmler B, Fraser CM (2002). Complete genome sequence and comparative analysis of the metabolically versatile *Pseudomonas putida* KT2440. Environ Microbiol.

[CR47] Nguyen TT, Youssef NH, McInerney MJ, Sabatini DA (2008). Rhamnolipid biosurfactant mixtures for environmental remediation. Water Res.

[CR48] Noordman WH, Janssen DB (2002). Rhamnolipid stimulates uptake of hydrophobic compounds by *Pseudomonas aeruginosa*. Appl Environ Microbiol.

[CR49] Ochsner UA, Fiechter A, Reiser J (1994). Isolation, characterization, and expression in *Escherichia coli* of the *Pseudomonas aeruginosa rhlAB* genes encoding a rhamnosyltransferase involved in rhamnolipid biosurfactant synthesis. J Biol Chem.

[CR50] Ochsner UA, Koch AK, Fiechter A, Reiser J (1994). Isolation and characterization of a regulatory gene affecting rhamnolipid biosurfactant synthesis in *Pseudomonas aeruginosa*. J Bacteriol.

[CR51] Ochsner UA, Reiser J (1995). Autoinducer-mediated regulation of rhamnolipid biosurfactant synthesis in *Pseudomonas aeruginosa*. Proc Natl Acad Sci U S A.

[CR52] Ochsner UA, Reiser J, Fiechter A, Witholt B (1995). Production of *Pseudomonas aeruginosa* rhamnolipid biosurfactants in heterologous hosts. Appl Environ Microbiol.

[CR53] Olvera C, Goldberg JB, Sánchez R, Soberón-Chávez G (1999). The *Pseudomonas aeruginosa algC* gene product participates in rhamnolipid biosynthesis. FEMS Microbiol Lett.

[CR54] Pearson JP, Pesci EC, Iglewski BH (1997). Roles of *Pseudomonas aeruginosa las* and *rhl quorum-sensing* systems in control of elastase and rhamnolipid biosynthesis genes. J Bacteriol.

[CR55] Patel RM, Desai AJ (1997). Surface-active properties of rhamnolipids from *Pseudomonas aeruginosa* GS3. J Basic Microbiol.

[CR56] Rahim R, Burrows LL, Monteiro MA, Perry MB, Lam JS (2000). Involvement of the *rml* locus in core oligosaccharide and O polysaccharide assembly in *Pseudomonas aeruginosa*. Microbiology.

[CR57] Rahim R, Ochsner UA, Olvera C, Graninger M, Messner P, Lam JS, Soberón-Chávez G (2001). Cloning and functional characterization of the *Pseudomonas aeruginosa rhlC* gene that encodes rhamnosyltransferase 2, an enzyme responsible for di-rhamnolipid biosynthesis. Mol Microbiol.

[CR58] Rehm BH, Mitsky TA, Steinbüchel A (2001). Role of fatty acid de novo biosynthesis in polyhydroxyalkanoic acid PHA and rhamnolipid synthesis by pseudomonads: establishment of the transacylase PhaG-mediated pathway for PHA biosynthesis in *Escherichia coli*. Appl Environ Microbiol.

[CR59] Reis RS, Pereira AG, Neves BC, Freire DMG (2011). Gene regulation of rhamnolipid production in *Pseudomonas aeruginosa*—a review. Bioresour Technol.

[CR60] Rosenau F, Isenhardt S, Gdynia A, Tielker D, Schmidt E, Tielen P, Schobert M, Jahn D, Wilhelm S, Jaeger KE (2010). Lipase LipC affects motility, biofilm formation and rhamnolipid production in *Pseudomonas aeruginosa*. FEMS Microbiol Lett.

[CR61] Salager JL, Morgan JC, Schechter RS, Wade WH, Vasquez E (1979). Optimum formulation of surfactant/water/oil systems for minimum interfacial tension or phase behavior. Soc Petrol Eng J.

[CR62] Sambrook J, Russell DW (2001). Molecular Cloning.

[CR63] Schenk T, Schuphan I, Schmidt B (1995). High-performance liquid-chromatographic determination of the rhamnolipids produced by *Pseudomonas aeruginosa*. J Chromatogr A.

[CR64] Simon R, Priefer U, Puhler A (1983). A broad host range mobilization system for in vivo genetic engineering: transposon mutagenesis in Gram negative bacteria. Nat Biotech.

[CR65] Soberón-Chávez G, Lépine F, Déziel E (2005). Production of rhamnolipids by *Pseudomonas aeruginosa*. Appl Microbiol Biotechnol.

[CR66] Sotirova A, Spasova D, Vasileva-Tonkova E, Galabova D (2009). Effects of rhamnolipid-biosurfactant on cell surface of *Pseudomonas aeruginosa*. Microbiol Res.

[CR67] Syldatk C, Lang S, Matulovic U, Wagner F (1985). Production of four interfacial active rhamnolipids from n-alkanes or glycerol by resting cells of *Pseudomonas* species DSM 2874. Z Naturforsch C.

[CR68] Syldatk C, Lang S, Wagner F, Wray V, Witte L (1985). Chemical and physical characteritation of four interfacial-active rhamnolipids from *Pseudomonas* spec. DSM 2874 grown on n-alkanes. Z Naturforsch C.

[CR69] Tiso T, Sabelhaus A, Behrens B, Wittgens A, Rosenau F, Hayen H, Blank LM (2016). Creating metabolic demand as an engineering strategy in *Pseudomonas putida*—rhamnolipid synthesis as an example. Metab Eng Commun.

[CR70] Toribio J, Escalante AE, Soberón-Chávez G (2010). Rhamnolipids: production in bacteria other than *Pseudomonas aeruginosa*. Eur J Lipid Sci Technol.

[CR71] Tremblay J, Richardson AP, Lépine F, Déziel E (2007). Self-produced extracellular stimuli modulate the *Pseudomonas aeruginosa* swarming motility behaviour. Environ Microbiol.

[CR72] Trummler K, Effenberger F, Syldatk C (2003). An integrated microbial/enzymatic process for production of rhamnolipids and L-+-rhamnose from rapeseed oil with *Pseudomonas* sp DSM 2874. Eur J Lipid Sci Technol.

[CR73] van Gennip M, Christensen LD, Alhede M, Phipps R, Jensen PØ, Christophersen L, Pamp SJ, Moser C, Mikkelsen PJ, Koh AY, Tolker-Nielsen T, Pier GB, Høiby N, Givskov M, Bjarnsholt T (2009). Inactivation of the *rhlA* gene in *Pseudomonas aeruginosa* prevents rhamnolipid production, disabling the protection against polymorphonuclear leukocytes. APMIS.

[CR74] Wang QH, Fang XD, Bai BJ, Liang XL, Shuler PJ, Goddard WA, Tang YC (2007). Engineering bacteria for production of rhamnolipid as an agent for enhanced oil recovery. Biotechnol Bioeng.

[CR75] Wilhelm S, Gdynia A, Tielen P, Rosenau F, Jaeger KE (2007). The autotransporter esterase EstA of *Pseudomonas aeruginosa* is required for rhamnolipid production, cell motility, and biofilm formation. J Bacteriol.

[CR76] Wilhelm S, Tommassen J, Jaeger KE (1999). A novel lipolytic enzyme located in the outer membrane of *Pseudomonas aeruginosa*. J Bacteriol.

[CR77] Williams P, Cámara M (2009). Quorum sensing and environmental adaptation in *Pseudomonas aeruginosa*: a tale of regulatory networks and multifunctional signal molecules. Curr Opin Microbiol.

[CR78] Winsor GL, Griffiths EJ, Lo R, Dhillon BK, Shay JA, Brinkman FS (2016). Enhanced annotations and features for comparing thousands of *Pseudomonas* genomes in the *Pseudomonas* genome database. Nucleic Acids Res.

[CR79] Winsor PA (1954). Solvent properties of amphiphilic compounds.

[CR80] Wittgens A, Tiso T, Arndt TT, Wenk P, Hemmerich J, Müller C, Wichmann R, Küpper B, Zwick M, Wilhelm S, Hausmann R, Syldatk C, Rosenau F, Blank LM (2011) Growth independent rhamnolipid production from glucose using the non-pathogenic *Pseudomonas putida* KT2440. Microb Cell Fact 10(80). doi:10.1186/1475-2859-10-80 10.1186/1475-2859-10-80 PMC3258213 21999513

[CR81] Zhang Y, Miller RM (1992). Enhanced octadecane dispersion and biodegradation by a *Pseudomonas* rhamnolipid surfactant biosurfactant. Appl Environ Microbiol.

[CR82] Zhang Y, Miller RM (1994). Effect of a *Pseudomonas* rhamnolipid biosurfactant on cell hydrophobicity and biodegradation of octadecane. Appl Environ Microbiol.

[CR83] Zhang Y, Miller RM (1995). Effect of rhamnolipid biosurfactant structure on solubilization and biodegradation of n-alkanes. Appl Environ Microbiol.

[CR84] Zhong H, Zeng GM, Liu JX, XM X, Yuan XZ, HY F, Huang GH, Liu ZF, Ding Y (2008). Adsorption of monorhamnolipid and dirhamnolipid on two *Pseudomonas aeruginosa* strains and the effect on cell surface hydrophobicity. Appl Microbiol Biotechnol.

[CR85] Zhu K, Rock CO (2008). RhlA converts β-hydroxyacyl-acyl carrier protein intermediates in fatty acid synthesis to the β-hydroxydecanoyl- β-hydroxydecanoate component of rhamnolipids in *Pseudomonas aeruginosa*. J Bacteriol.

